# Defocused travelling fringes in a scanning triple-Laue X-ray interferometry setup

**DOI:** 10.1107/S1600576721007962

**Published:** 2021-09-13

**Authors:** C. P. Sasso, G. Mana, E. Massa

**Affiliations:** a INRIM – Istituto Nazionale di Ricerca Metrologica, strada delle cacce 91, 10135 Torino, Italy; b UNITO – Università di Torino, Dipartimento di Fisica, via Pietro Giuria 1, 10125 Torino, Italy

**Keywords:** X-ray interferometry, dynamical theory of X-ray diffraction, X-ray crystal density, Si lattice parameter

## Abstract

The measurement of the silicon lattice parameter by a separate-crystal triple-Laue X-ray interferometer is a key step for the realization of the kilogram by counting atoms. Since the measurement accuracy is approaching nine significant digits, a reliable model of the interferometer operation is required to quantify or exclude systematic errors. This paper investigates both analytically and experimentally the effect of the defocus (the difference between the splitter-to-mirror and analyser-to-mirror distances) on the phase of the interference fringes and the measurement of the lattice parameter.

## Abstract 2

This paper investigates both analytically and experimentally the effect of the defocus (the difference between the splitter-to-mirror and analyser-to-mirror distances) on the phase of the travelling fringes in a separate-crystal triple-Laue X-ray interferometer.

## 1. Introduction   

The measurement of the silicon lattice parameter at optical wavelengths by scanning X-ray interferometry opened a broad field of metrological and science applications. In addition to realizing the metre at atomic length scales (Basile *et al.*, 2000 ▸), to determining the Avogadro constant (Fujii *et al.*, 2018 ▸), and, nowadays, to realizing the kilogram from the Planck constant *h*, it was instrumental in the determination of the *h*/*m*_n_ ratio (Krueger *et al.*, 1998 ▸, 1999 ▸) and allowed the wavelength of X- and γ-rays to be referred to the metre. These links resulted in improved measurements of the deuteron binding energy and neutron mass *m*_n_ (Greene *et al.*, 1986 ▸; Kessler *et al.*, 1999 ▸) and the most accurate test of the Planck–Einstein identity *h*ν = *mc* ^2^ (Rainville *et al.*, 2005 ▸).

In 2010 and 2014, we completed measurements of the lattice parameter of an ^28^Si crystal in order to determine the Avogadro constant and, more recently, to realize the kilogram by counting atoms (Massa *et al.*, 2011 ▸, 2015 ▸). The assessment and further improvements of the measurement accuracy, approaching nine significant digits, require a reliable model of the interferometer operation to quantify or exclude parasitic contributions to the fringe phase originated by unavoidable aberrations.

The operation theory of a triple-Laue interferometer was developed by Bonse & Hart (1965 ▸), Bonse & te Kaat (1971 ▸), Bauspiess *et al.* (1976 ▸) and Bonse & Graeff (1977 ▸) and refined, with particular emphasis on the aberration effects on the fringe phase, by Vittone & Zosi (1994 ▸), Mana & Vittone (1997*a* ▸,*b* ▸) and Mana & Montanari (2004 ▸). In the present paper, we report an experimental verification of the dynamical-theory calculation of the out-of-focus effect on the fringe phase.

The paper is organized as follows. After a short description of the experimental setup, in Section 3 we sketch the dynamical theory of the interferometer operation. Section 4 reports the numerical calculation of the defocus effect on the fringe phase for the interferometer cut from the ^28^Si crystal whose lattice parameter was an input datum for the determination of the Avogadro constant. All the computations were carried out with the aid of *Mathematica* (Wolfram Research, 2020 ▸). The relevant notebook is given as supplementary material. The measured values of the fringe-phase sensitivity to the defocus are given in Section 5.

## 2. X-ray interferometry   

**Figure 1:**
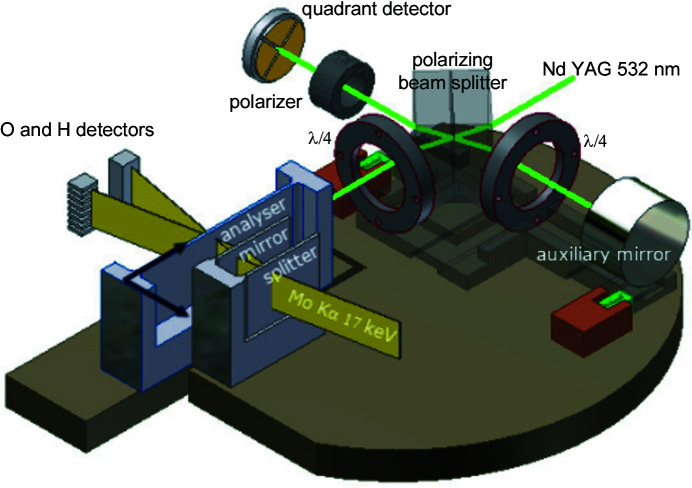
INRIM’s combined X-ray and optical interferometer. The analyser displacement and the pitch and yaw angles are measured by laser interferometry and differential wavefront sensing. The transverse displacements (horizontal and vertical) are measured via capacitive sensors (not shown in the figure). The arrows indicate the positive directions of the axial and out-of-focus displacements.

As shown in Fig. 1 ▸, our X-ray interferometer splits and recombines, by a separate analyser crystal, Mo *K*α_1_ X-rays by Laue diffraction in perfect Si crystals. X rays are collimated to about 0.25 mrad divergence by means of a slit (not shown in the figure) placed in front of the interferometer. The splitter, mirror and analyser operate in symmetric geometry, where the {220} diffracting planes are perpendicular to the crystals’ surfaces. When moving the analyser orthogonally to the diffracting planes, the interfering beams are phase shifted and travelling interference fringes are observed, the period being the plane spacing, *d*_220_ ≃ 192 pm. To ensure temperature uniformity and stability and to eliminate the adverse influence of the refractive index of air, the apparatus is hosted in a (passive) thermovacuum chamber.

Detailed descriptions of the experimental apparatus are given elsewhere (Bergamin *et al.*, 1993 ▸, 2003 ▸; Ferroglio *et al.*, 2008 ▸; Massa *et al.*, 2011 ▸, 2015 ▸, 2020 ▸). The analyser is displaced using a guide where an L-shaped carriage slides on a quasi-optical rail. An active platform with three piezoelectric legs rests on the carriage. Each leg expands vertically and shears in the two transverse directions, thus positioning the analyser over six degrees of freedom to atomic-scale accuracy. The analyser displacement, parasitic rotations (pitch, yaw and roll) and transverse motions (horizontal and vertical) are measured via laser interferometry, differential wavefront sensing and capacitive transducers. Feedback loops provide (axial) picometre positioning, nanoradian alignment and axial movements with nanometre straightness.

## 3. Dynamical theory of the interferometer operation   

The solutions of the Takagi–Taupin equations for the propagation of X-rays in perfect crystals and triple-Laue interferometers are given by Mana & Vittone (1997*a* ▸) and Mana & Montanari (2004 ▸). The crystal field resembles a quantum two-level system. In a two-dimensional model, these authors define the Hilbert space 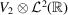, where *V*_2_ is a two-dimensional vector space (the space of the dispersion-surface branches) and 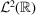 is the space of the square-integrable functions.

**Figure 2:**
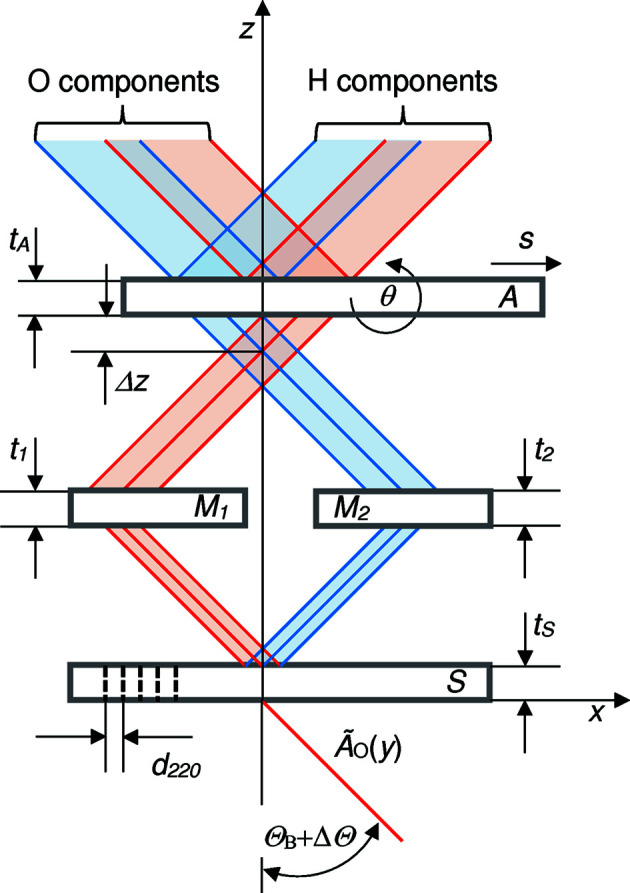
Two-dimensional model of a symmetrical LLL interferometer. S splitter, M1 and M2 mirrors, A analyser. Red and blue rays indicated paths 1 and 2, respectively. The *x* axis is orthogonal to the diffracting planes. Θ_B_ is the Bragg angle, Δ*z* the defocus (positive if the analyser moves towards the negative **z** direction), θ the analyser misalignment and *s* the analyser displacement (positive if the analyser moves towards the positive **x** direction).

With coherent and monochromatic illumination and omission or rearrangement of common constant and phase terms, the reciprocal-space representations of the waves that leave the interferometer after crossing it along paths 1 and 2 are 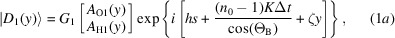


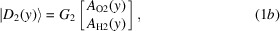
where 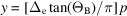 is the deviation parameter, ζ = 2πΔ*z*/Δ_e_ is the dimensionless defocus, *p* is the variable conjugate to *x*, 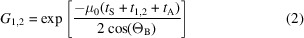
accounts for the photoelectric absorption, 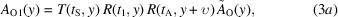


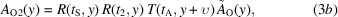


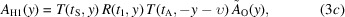


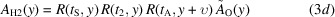
are the complex amplitudes of the O and H Bloch waves 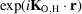, 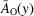 is the reciprocal-space representation of the amplitude of the incoming Bloch wave, 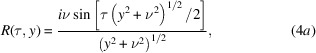


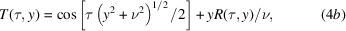
are the scattering amplitudes, and τ = 2π*t*/Δ_e_ is the dimensionless crystal thickness. The indexes β = σ, π indicating the polarization states parallel and orthogonal to the reflection plane have been omitted. The symbols that are not defined above are given in Fig. 2 ▸ and Table 1 ▸. With the convention adopted, the displacement *s* and defocus Δ*z* are positive in the **x** and −**z** directions, respectively.

On the analyser surface, the direct- and reciprocal-space representations of the complex amplitude of the incoming Bloch wave are 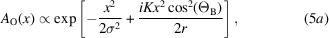


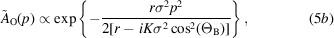
where *K* = |**K**_O,H_| is the wavenumber, σ is the beam radius and 1/*r* is the wavefront curvature.

Free propagation leads to the spatial separation of the O and H components of (1*a*) and (1*b*)[Disp-formula fd1b] into two localized single-component waves, which overlap and interfere. Detectors do not resolve the interference pattern but measure the total photon fluxes. Consequently, an integration is necessary to describe the detected signals: 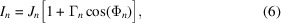
where *n* = O, H.

Owing the limited transverse extensions of the interfering beams and large detectors, we set an infinite aperture and carry out the integration in the reciprocal space. Finally, since photons produced by conventional X-ray sources can have any polarization, with equal probability, we add the σ and π polarizations incoherently. Therefore, in (6), 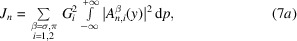


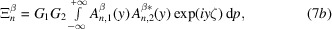


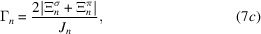


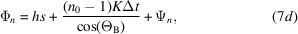


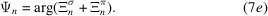
According to (7), the crystal displacement *s* gives rise to travelling fringes whose period is the spacing *d*_220_ = 2π/*h* of the diffracting planes. In the reciprocal space, the defocus (the difference between the splitter-to-mirror and analyser-to-mirror distances) shifts by 2π*y*Δ*z*/Δ_e_ the phase of the plane wave components travelling along paths 1 and 2. In the real space, it shears the interfering beams by 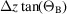. With a perfect geometry (that is, *t*_S_ = *t*_A_, *t*_1_ = *t*_2_ and υ = θΔ_e_/*d*_220_ = 0) and 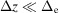, the symmetries 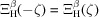 and 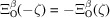 imply that the defocus has no effect on the phase of the H-beam fringes and changes linearly those of the O beam (Mana & Vittone, 1997*b* ▸).

## 4. Numerical simulation   

**Figure 3:**
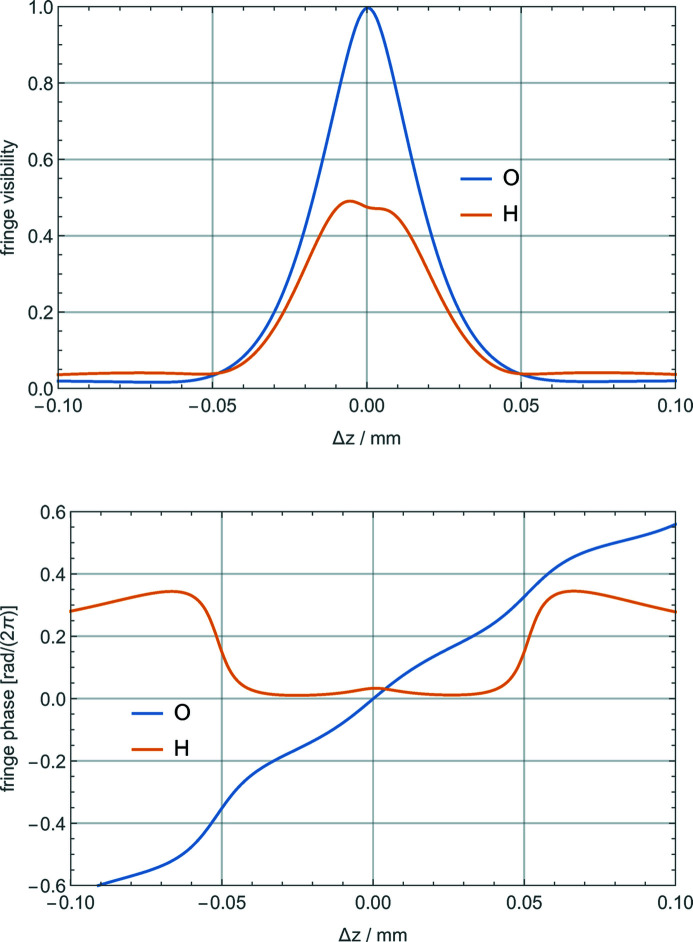
Visibility and phase excess Ψ_O,H_ of the O and H travelling fringes versus the interferometer defocus Δ*z*, which is positive if the analyser moves towards the negative **z** direction (see Figs. 1 ▸ and 2 ▸).

By using the formalism developed by Mana & Vittone (1997*a* ▸) and Mana & Montanari (2004 ▸) and outlined in Section 3, we calculated the visibility and phase of the travelling fringes as a function of the defocus. The parameters used, which refer to the interferometer used to determine the lattice parameter of ^28^Si (Massa *et al.*, 2011 ▸, 2015 ▸), are listed in Table 2 ▸. The visibility loss and phase shift are shown in Fig. 3 ▸. For large defocuses, the phase shift is sensitive to the exact interferometer geometry and operation: when the phasor representing the fringes is around the origin, a phase jump occurs. In Fig. 3 ▸, this bypass occurs for the fringes belonging to the reflected beam. Also, no phase measurement is possible without fringe visibility.

The interferometer defocus contributes to the travelling-fringe phase as 2π*c*_*n*_Δ*z*, which is valid if 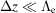, where Λ_e_ is the *Pendellösung* length. As shown in Fig. 3 ▸, imperfections break the visibility and phase symmetries and change the sensitivities to the defocus. To take the interferometer geometry’s uncertainty into account, we evaluated the phase sensitivities to the defocus, *c*_O_ and *c*_H_, by a Monte Carlo simulation. Table 2 ▸ gives the simulation parameters and the standard deviations of the normal distributions from which we repeatedly sampled the crystal thicknesses, defocus and analyser misalignment. They have been set according to the experimental capabilities to control the interferometer geometry and alignment. The means and standard deviations of the Monte Carlo populations are *c*_O_ = 0.0082 (20) µm^−1^ (O beam) and *c*_H_ = 0.0004 (20) µm^−1^ (H beam).

**Figure 4:**
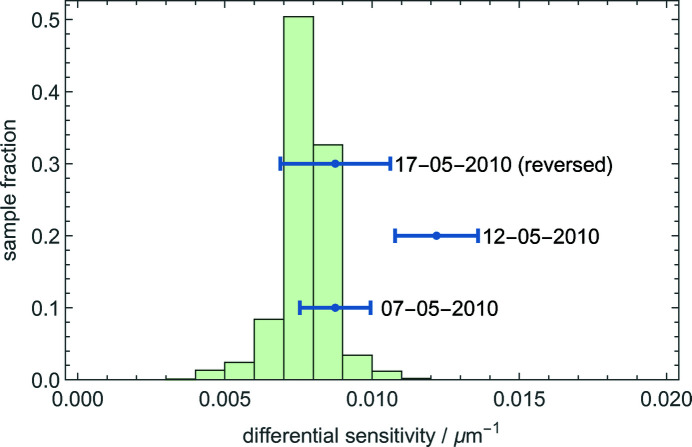
Results of the Monte Carlo calculation of the difference between the phase sensitivities of the O and H travelling fringes. The interferometer parameters and associated uncertainties given in Table 2 ▸. Blue: observed values of the differential sensitivity *c*_O_ − *c*_H_; the lines represent the standard uncertainties.

In the next section we will explain that the observable quantity is the differential sensitivity Δ*c* = *c*_O_ − *c*_H_, whose frequencies of occurrence in the Monte Carlo population are shown in Fig. 4 ▸. The population mean and standard deviation are Δ*c* = 0.0078 (9) µm^−1^; the reduced uncertainty follows by the correlation between *c*_O_ and *c*_H_.

## 5. Experimental test   

For the experimental verification of the these predictions, we mined useful data from the archive of the lattice parameter measurements carried out in 2010. At that time, to countercheck a previous measurement of the angle between the analyser front mirror and the diffracting planes (Bergamin *et al.*, 1999 ▸; Sasso *et al.*, 2021 ▸), we defocused the interferometer by moving the analyser transversely, in a direction opposite the *z* axis in Fig. 2 ▸, and recorded the interferometer signals before and after the displacement. Because of the supporting platform’s small operating range, the defocus was limited to 3.20 (15) µm.

A feedback loop, relying on the laser interferometer’s signals, locked to zero the axial displacement and the pitch and yaw rotations of the analyser (to within 1 pm and 1 nrad). In this way, we ensured that the translation occurred in the plane of the front mirror, which is ideally parallel to the diffracting planes.

However, a miscut angle makes the front mirror slightly misaligned and, therefore, the defocus shows a small axial component (Sasso *et al.*, 2021 ▸). For this reason, the only quantity experimentally observable is the difference between the phase sensitivities of the travelling fringes observed in the O and H beams. In fact, any axial displacement originates a common mode phase that can be eliminated by differentiation of the phase change in the O and H branches.

The vertical and horizontal offsets between the laser and X-ray beams were nullified to avoid differential Abbe errors. In the O beam, the interference pattern is imaged into a multi-anode photomultiplier through a stack of eight NaI(Tl) scintillators and the virtual pixel having no vertical offset is identified. In the H beam, we imaged the whole vertical extension of the interference pattern and nullified the offset by windowing.

**Figure 5:**
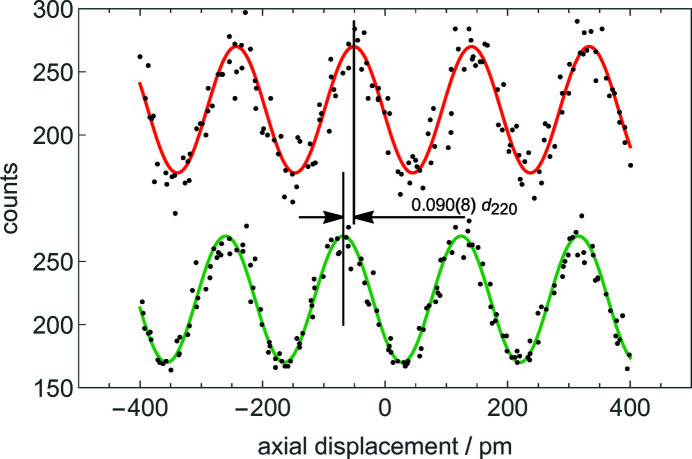
H beam. Scans of the X-ray fringes (H beam) before (red) and after (green) a positive defocus of 3.20 (15) µm (see Figs. 1 ▸ and 2 ▸). The dots are the X-photons counted in 100 ms. The solid lines are the best-fit sinusoids approximating the data. The observed phase difference is 0.090 (8) *d*_220_ (see the first two data points in Fig. 6 ▸).

Since it was not possible to eliminate the drift between the optical and X-ray fringes, we implemented a modulation–demodulation strategy. We repeatedly defocused the interferometer and the two – optical and X-ray – signals were sampled before and after each defocusing. As shown in Fig. 5 ▸, the phases of the travelling X-ray fringes before and after defocus were recovered by least-squares estimations via the model 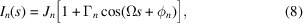
where *J*_*n*_, Γ_*n*_, Ω and ϕ_*n*_ are unknown parameters to be determined, and *n* = O, H. ϕ_O_ − ϕ_H_ = 2πΔ*c*Δ*z* is the phase difference that we seek with the aim of verifying the theoretical Δ*c* prediction, and the constraints Γ_*n*_ > 0 and Ω > 0 were applied (Bergamin *et al.*, 1991 ▸). The displacement *s* is positive when the analyser moves towards the positive **x** direction (see Figs. 1 ▸ and 2 ▸).

**Figure 6:**
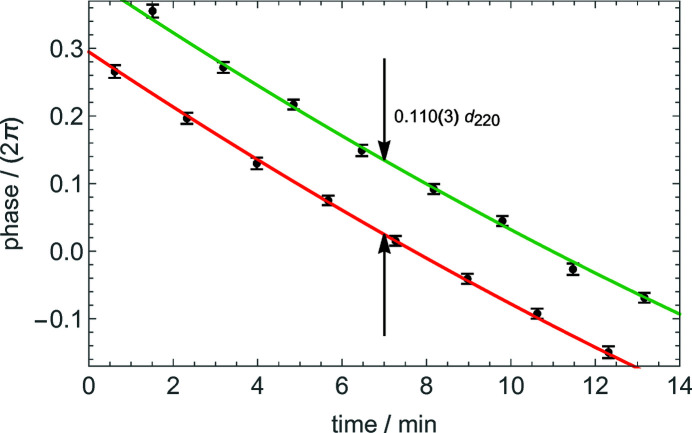
H beam. Phases of the X-ray fringes measured before (red) and after (green) a positive defocus of 3.20 (15) µm (see Figs. 1 ▸ and 2 ▸). The dots are the measured phases (see Fig. 5 ▸). The bars are the associated standard uncertainties. The phase difference, 0.110 (3) *d*_220_, is not null because of the analyser miscut angle.

**Figure 7:**
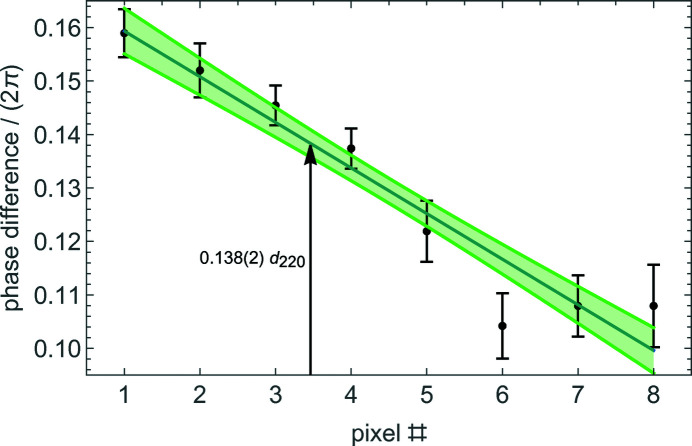
O beam. Phase differences between the defocused – 3.20 (15) µm – and focused travelling fringes versus the detector pixels. The bars are the associated standard uncertainties. The solid line is the line approximating the data; the filled area indicates the standard deviation. The phase difference at the 3.46 virtual pixel having the same residual vertical offset as the H beam is 0.138 (2) *d*_220_.

Next, as shown in Fig. 6 ▸, the drift was identified and subtracted by fitting the phases of the X-ray fringes with polynomials differing only by the sought phase difference. For the O beam, we calculated the phase difference between the defocused and focused fringes at the virtual pixel having the same residual vertical offset as the H beam. The result is shown in Fig. 7 ▸. The difference between the phase sensitivities to the defocus of the O and H fringes obtained from the data shown in Figs. 6 ▸ and 7 ▸ is 0.028 (4)/3.20 (15) µm^−1^ = 0.0088 (12) µm^−1^. The phase gradient in Fig. 7 ▸ is due to the second derivative of the residual angular instability of the laser interferometer. This instability is copied by the feedback loops into the analyser misalignment, and the nonlinearity is not removed by the modulation–demodulation process.

To compare the predicted difference against the observed ones, we chose the positive signs of the analyser displacement and defocus in the same way in both the interferometer model (1*b*)[Disp-formula fd1b] and the analysis of the experimental data (8). The results are given in Table 3 ▸ and shown in Fig. 4 ▸. The measurements were carried out on 7, 12 and 17 May 2010. The first two measurements were carried out at two different axial positions of the analyser, spaced by about 30 mm. We carried out the last after the analyser’s reversal, which exchanged the entrance and exit surfaces.

## 6. Conclusions   

A study of the signals from a combined X-ray and optical interferometer revealed a satisfactory agreement between the observed and predicted phase shifts of the travelling X-ray fringes due to the defocus. This result is directly applicable to assessing the measured values of the ^28^Si lattice parameter and confirms that micrometre changes of focus were irrelevant to the error budget of our 5 cm scan (Massa *et al.*, 2011 ▸, 2015 ▸). In fact, a very large parasitic defocus of 10 µm associated with an *s* = 5 cm travel of the analyser will cause, in the worst O-beam case, a fractional phase error of *c*_O_ *d*_220_/*s* ≃ 3 × 10^−10^.

However, if a 1 nm m^−1^ fractional accuracy is the aim, measurements over submillimetre scans must consider the changes of focus seriously, for instance, those due to an insufficient flatness of the analyser surface (Andreas & Kuetgens, 2020 ▸).

Our result is also applicable to the completeness of the dynamical theory of X-ray diffraction in a perfect crystal, although this time we were not aiming to testing the dynamical-theory predictions. The unexplained discrepancy of the 2010-05-12 datum might be ascribed to an insufficient control of the interferometer operation. Future experiments with larger and better-calibrated defocus are feasible and might put our conclusions on a still safer footing.

## Supplementary Material

Mathematica notebook with the simulation routines (application/vnd.wolfram.mathematica). DOI: 10.1107/S1600576721007962/vh5143sup1.txt

## Figures and Tables

**Table 1:** List of symbols

{\bf h} = 2\pi\hat{{\bf x}}/d_{220}	Reciprocal vector
*d*_220_	Diffracting plane spacing
*s*	Analyser displacement
*t*_S_, *t*_1_, *t*_2_, *t*_A_	Crystal thicknesses (splitter, mirrors and analyser)
Δ*t* = *t*_1_ − *t*_2_	Differential mirror thickness
τ = 2π*t*/Δ_e_	Dimensionless crystal thickness
Δ*z*	Defocus
ζ = 2πΔ*z*/Δ_e_	Dimensionless defocus
θ	Analyser misalignment
**K**_H_ = **K**_O_ + **h**	Wavevectors of the O and H Bloch waves
2**h** · **K**_O_ = *h* ^2^	Bragg’s law
\sin(\Theta_{\rm B}) = -{({{\bf K}_{\rm O}\!\!\cdot\!{\bf h}}) / {K_{\rm O}h}}	Bragg angle
\Delta\Theta = {{p} / [{K\cos(\Theta_{\rm B})}]}	Plane-wave deviation from Bragg alignment
χ_0,*h*_	Fourier components of the electric susceptibilities
n_{0} = 1+\Re(\chi_{0})/2	Refractive index
\mu_{0} = \Im(\chi_{0})K	Absorption coefficient
\kappa = \arg(\chi_{h})	
\nu = \exp({\rm{i}}\kappa)	
\Delta_{\rm e} = \lambda\cos(\Theta_{B})/|\chi_{h}|	*Pendellösung* length
y = \Delta_{\rm e}\tan(\Theta_{\rm B})p/\pi = ΔΘΔ_e_/*d*_220_	Deviation parameter
υ = θΔ_e_/*d*_220_	Deviation-parameter shift (analyser misalignment)

**Table 2:** Parameters used in the numerical computations The dielectric susceptibilities are from Stepanov’s X-ray server (Stepanov, 2004 ▸). For the π polarization, we have included the \cos(2\Theta_{\rm B}) factor in the *h* component of the electric susceptibility. The crystal thicknesses are the mean values. The values in parentheses are the standard deviations of the variables assigned randomly in the Monte Carlo simulation

χ_0_ = −3.1745 × 10^−6^ + 1.6060 × 10^−8^ *i*	
\chi_{h}^{\sigma} = 1.9210\times 10^{-6}-1.5497\times 10^{-8}i	
\chi_{h}^{\pi} = (1.7899\times 10^{-6}-1.4345\times 10^{-8}i)\cos(2 \Theta_{\rm B})	
λ = 0.0709317 nm	*d*_220_ = 192.014 pm
*t*_S_ = 1.196 (2) mm	*t*_A_ = 1.197 (2) mm
*t*_1_ = 1.193 (2) mm	*t*_2_ = 1.193 (2) mm
Δ*z* = 0(4) µm	θ = 0(1) µrad
\Delta_{\rm e}^{\sigma} = 36.29 µm	\Delta_{\rm e}^{\pi} = 41.80 µm
μ_0_ = 1.423 × 10^−3^ µm^−1^	*n*_0_ − 1 = 1.587 × 10^−6^
κ_π_ ≃ κ_σ_ ≃ π	ν_π_ ≃ ν_σ_ ≃ −1

**Table 3:** Measured differences between the phase sensitivities to the defocus of the O and H fringes The expected value is 0.0078 (9) µm^−1^.

Date	Differential sensitivity (µm^−1^)
07-05-2010	0.0088 (12)
12-05-2010	0.0122 (14)
17-05-2010	0.0088 (19)
